# Variants in *ADIPOQ* gene are linked to adiponectin levels and lung function in young males independent of obesity

**DOI:** 10.1371/journal.pone.0225662

**Published:** 2020-01-24

**Authors:** Andria Christodoulou, Despo Ierodiakonou, Awoyemi A. Awofala, Michael Petrou, Stefanos N. Kales, David C. Christiani, Christos S. Mantzoros, Costas A. Christophi

**Affiliations:** 1 Cyprus International Institute for Environmental and Public Health, Cyprus University of Technology, Limassol, Cyprus; 2 Department of Social Medicine Faculty of Medicine, University of Crete, Crete, Greece; 3 Department of Biological Sciences, Tai Solarin University of Education, Ijagun, Ogun State, Nigeria; 4 Cyprus Anti-doping Authority, Nicosia, Cyprus; 5 Department of Environmental Health, Harvard T.H. Chan School of Public Health, Boston, MA, United States of America; 6 Cambridge Health Alliance, Harvard Medical School, Cambridge, MA, United States of America; 7 Department of Medicine, Beth Israel Deaconess Medical Center and Harvard Medical School, Boston, MA, United States of America; East Tennessee State University, UNITED STATES

## Abstract

**Background:**

Obesity is a major risk factor for many chronic diseases, including reduced lung function. The role of polymorphisms of the adiponectin gene, though linked with cardiometabolic consequences of obesity, has not been studied in relation to lung function.

**Objectives:**

The aim of this study is to examine polymorphisms in the *ADIPOQ*, *ADIPOR1*, and *ADIPOR2* genes in relation to adiponectin serum levels, BMI, and adiposity in 18-year old Cypriot males, as well as determine whether BMI, adipokines levels and polymorphisms in adipokine related genes are associated with lung function levels.

**Results:**

From the participants, 8% were classified as obese, 22% as overweight, and the remaining 71% as normal. We found that rs266729 and rs1501299 in *ADIPOQ* and rs10920531 in *ADIPOR1* were significantly associated with serum adiponectin levels, after adjusting for ever smoking. In addition, there was an overall significant increase in FEV_1_% predicted with increasing BMI (β = 0.53, 95% CI: 0.27, 0.78) and in FVC % predicted (β = 1.02, 95% CI: 0.73, 1.30). There was also a decrease in FEV_1_/FVC with increasing BMI (β = -0.53, 95% CI: -0.71, -0.35). Finally, rs1501299 was associated with lung function measures.

**Discussion:**

Functional variants in the ADIPOQ gene were linked with lung function in young males. Further studies should concentrate on the role of adipokines on lung function which may direct novel therapeutic approaches.

## Introduction

Obesity is a major public health concern and worldwide 150 million adults and 15 million children are obese [1]. Although in the past obesity was considered a problem only for high income countries, overweight and obesity are nowadays increasing dramatically in low- and middle-income countries as well, particularly in urban settings [2]. In Cyprus, a small European island in the Eastern Mediterranean region, it has reached epidemic proportions with data from a cross-sectional study suggesting that 36% and 28% of the adult population are overweight and obese, respectively [3].

Obesity is a disorder which is a major risk factor for many other chronic diseases, including type 2 diabetes, cardiovascular disease, and cancer, as well as for lung diseases and reduced lung function [4–7]. Different studies have shown associations between increased Body Mass Index (BMI) and asthma [8], and evidence suggests that obese individuals aged less than 18 years old show a reduction in lung volume and capacity as compared to healthy individuals [9]. Obesity causes deleterious effects on lung volume and capacity in children and adolescents, mainly by reducing functional residual capacity, expiratory reserve volume, and residual volume [10]. However, initial increases in weight increase lung function among the non-obese. These increases reflect an increase in muscle force. Afterwards, further increases in body mass represent obesity, and as a consequence lung function, decreases [11]. Additionally, among young males’ the lungs grow up to the age of 21, justifying the fact that an increase in BMI and thus lung function is usually associated with the natural growth of lungs rather than obesity.

Adipose tissue produces adipokines which are proteins that regulate inflammation and metabolism in an autocrine, paracrine, and systemic manner [12,13]. Adiponectin in particular is an adipokine, which is associated with systemic anti-inflammatory effects, and serum adiponectin concentrations are known to be reduced in obese and asthmatic individuals [14]. Adiponectin and its receptors are expressed on multiple cell types in the lung and a recent study suggested that lower serum adiponectin concentrations may be associated with lower lung function in young adults, independent of obesity [15].

Adiponectin cellular signaling is mediated by two adiponectin receptors, namely *ADIPOR1* (located on chromosome 1q32) and *ADIPOR2* (located on chromosome 12p13.33) [16]. Adiponectin levels are in part determined by genetic influences, with a recent study reporting heritability estimates of 51% [17], and they are encoded by the gene *ADIPOQ*, located on chromosome 3q27. As adiponectin is inversely associated with BMI and other measures of adiposity [13] the adiponectin related genes are considered important candidate genes for being related with obesity phenotypes, and thus could help in unraveling the genetic underpinnings of obesity. Although in various studies the polymorphisms of the ligand adiponectin gene, *ADIPOQ*, have been linked with cardiometabolic consequences of obesity, their role in lung function has not been studied yet.

The primary goal of this study was to examine the polymorphisms in *ADIPOQ*, *ADIPOR1*, and *ADIPOR2* in relation to adiponectin serum levels and adiposity in an 18-year old male population of Cyprus. Our secondary aim was to examine whether lung function levels in young males vary in association with BMI, adipokines’ levels, and polymorphisms in adipokine related genes.

## Methods

### Study participants

The Cyprus Metabolism Study included 1,056 healthy 18-year old male individuals, recruited on a voluntary basis during the July 2006 and July 2007 enrolment cycles of the Cyprus Army. This sample included participants from all Cypriot regions and socioeconomic levels, recruited while performing their two-year mandatory service in the Cyprus Army, and it is considered representative of the 18-year old male population of Cyprus. Exclusion criteria included health reasons for which a person was found unable to participate in the military service, including most chronic diseases. The study had approval by the Cyprus Bioethics Committee and the Institutional Review Board (IRB) of the Harvard T.H. Chan School of Public Health; all participants were informed about the study before enrolling and signed an informed consent.

### Clinical and other measurements

Participants underwent detailed clinical investigations. In brief, anthropometric parameters, including height, weight, waist circumference, hip circumference, skinfolds, and body fat were measured in all subjects according to standard protocols by trained personnel. BMI was calculated as weight in kilograms divided by the square of height in meters. Waist-to-height ratio was calculated as waist circumference in centimeters divided by height in centimeters. Information on ever smoking was collected using standard questionnaires together with demographic information, medical history, physical activity, and other. Fasting blood samples were drawn to assay the levels of serum adiponectin *(*μg/ml) using a standard immunoassay protocol. Spirometry was performed in 416 participants only and lung capacity was evaluated using a hand-held Micro-Spirometer Vitalograph 2120 in accordance to ATS/NIOSH guidelines. Participants were first instructed by trained personnel on how to use the spirometer and then three attempts were made keeping the best one.

### Selection and genotyping of SNPs

Twenty SNPs including 5 *ADIPOQ* SNPs (rs266729, rs822395, rs822396, rs1501299, and rs2241766), 5 *ADIPOR1* SNPs (rs2232853, rs12733285, rs1342387, rs7539542 and rs10920531), and 10 *ADIPOR2* SNPs (rs1029629, rs7975600, rs11612383, rs1058322, rs11061973, rs2108642, rs767870, rs12342, rs1044471, and rs7294540) were selected a priori. These SNPs were chosen based on the following criteria: (1) they capture variations in the major blocks in each gene, (2) they are functionally relevant and have been studied by others in relation to adiponectin levels or diseases, such as diabetes, metabolic and insulin resistance syndromes [16–20], and (3) they have a minor allele frequency greater than 10% in European individuals in the HapMap database. SNPs were genotyped with the Taqman/MALDI-TOF SNP allelic discrimination using a standard protocol. A total of 9.4% of samples were genotyped in duplicate and showed 98.8% concordance. The overall genotyping success rate was 99.0%.

### Statistical analysis

Continuous characteristics are presented as mean ± SD and compared among groups using the one-way analysis of variance technique, whereas categorical variables are presented as frequency (%) and compared among groups using the chi-square test of independence. BMI was used to classify individuals into obese (BMI ≥ 30 kg/m^2^), overweight (25 kg/m^2^ ≤ BMI < 30 kg/m^2^) and normal/underweight (reference weight) (BMI<25 kg/m^2^) categories. Linear regression models were used to assess the effect of genetic variation in the adiponectin-related genes on adiponectin levels and BMI after adjusting for smoking as well as with lung function. Results are reported as beta coefficients with the corresponding 95% confidence interval (CI). Similarly, logistic regression models were utilized to estimate the impact of genetic variations in the adiponectin-related genes on the probability of being overweight or obese, after adjusting for smoking, and results are reported as OR (95% CI). Dominant models were used which grouped the heterozygous with the homozygous for the minor allele. The referent genotype was selected to be the most common homozygous genotype. Haploview was used to determine the linkage disequilibrium (LD) between SNPs and departure from Hardy-Weinberg equilibrium was assessed using an exact test. In addition, linear regression models were used to assess the association of BMI and adiponectin serum levels with lung function after adjusting for smoking. Statistical analysis was performed using SAS software Version 9.3 (SAS Inc., Cary, NC, USA) and all tests performed were two-sided with P<0.05 indicating statistical significance. Bonferroni correction method was not used because in this hypothesis driven study selection of included SNPs of candidate genes was based on known functionality and previous findings of associations of SNPs with adiponectin levels or metabolic diseases.

## Results

Out of the 1,056 participants in the study 1,043 had available BMI information. Of them, 7.8% (n = 81) were classified as obese, 21.7% (n = 226) as overweight, and the remaining 70.6% (n = 736) as normal, which was the reference group (Table 1). The mean age of the participants was 18.4 ± 0.6 years and 40.8% of them were smokers. Obese participants had worse anthropometric measures than overweight and reference weight individuals and they were more likely to be smokers (p = 0.02). Furthermore, obese participants had similar adiponectin levels as overweight individuals but differed significantly from the reference weight individuals (p <0.01).

**Table 1:** Characteristics of participants by obesity group.

	All	Obese	Overweight	Normal	*P*-value
N	1043	81	226	736	
Age (years)	18.4 ± 0.6	18.4 ± 0.4	18.4 ± 0.6	18.4 ± 0.6	0.78
BMI (Kg/m^2^)	23.5 ± 4.1	33.2 ± 2.7	26.9 ± 1.4	21.4 ± 2.1	<0.01
Waist circumference (cm)	81.7 ± 10.7	105.4 ± 7.4	90.1 ± 6.1	76.6 ± 5.9	<0.01
Waist-to-height ratio	0.47 ± 0.06	0.60 ± 0.04	0.51 ± 0.03	0.44 ± 0.03	<0.01
Sums of skinfolds (cm)	50.9 ± 24.2	105.0 ± 21.6	67.7 ± 18.6	40.0 ± 12.6	<0.01
Fat percentage	12.9 ± 6.7	27.1 ± 4.9	18.0 ± 4.0	9.7 ± 3.8	<0.01
Smokers—n (%)	419 (40.8)	45 (55.6)	90 (40.7)	284 (39.2)	0.02
Adiponectin (μg/mL)	7.9 ± 3.8	6.8 ± 2.8	6.9 ± 3.6	8.3 ± 3.9	<0.01

The data for continuous variables is mean ± SD. The data for categorical variable is shown as n (%).

The location, genotypic frequencies, and the major and minor alleles of the SNPs selected for the three adiponectin-related genes are described in S1 Table while the results for the linkage disequilibrium (LD) of SNPs are shown in S1 Fig. The genotypes of all the SNPs were in Hardy-Weinberg equilibrium in each group (p>0.05). In addition, the minor allele frequencies of the SNPs were virtually identical to those reported in the HapMap database for Central European (CEU) population.

### Associations with BMI and adiposity

SNPs rs266729 and rs1501299 in *ADIPOQ* and rs10920531 in *ADIPOR1* were significantly associated with serum adiponectin levels, after adjusting for smoking (β and (95% CI): -0.58 (-1.09, -0.07), 0.60 (0.10, 1.10), and 0.57 (0.05, 1.09), with p-values 0.03, 0.02, and 0.03, respectively (Table 2)). These associations were not altered when BMI was added as a covariate to the corresponding models (S2 Table). In addition, there were no significant associations of SNP with BMI (Table 2) or with the probability of being overweight or obese (Table 3), though rs266729 in *ADIPOQ* and rs7975600 in *ADIPOR2* had a borderline significant association with the probability of being overweight (OR = 1.31, 95% CI: 0.95, 1.79, p = 0.10 and OR = 0.74, 95% CI: 0.51, 1.06, p = 0.10, respectively) and rs2232853 with the probability of being obese (OR = 1.61, 95% CI: 0.99, 2.61, p = 0.05). We further investigated whether there was an association between the SNPs and other anthropometric indices, such as waist circumference, waist-to-height ratio, fat percentage, and sum of skinfolds. Our results showed that none of the SNPs included was significantly associated with any of these anthropometric indices (S3 Table).

**Table 2:** Associations of genetic variants with adiponectin levels and Body Mass Index.

				Adiponectin (μg/mL)		BMI (kg/m^2^)	
SNP	Chr.	Gene	M/m	β (95% CI)	*P-value*	β (95% CI)	*P-value*
rs266729	3	*ADIPOQ*	C/G	-0.58 (-1.09, -0.07)	0.03	0.27 (-0.26, 0.81)	0.32
rs822395	3	*ADIPOQ*	A/C	0.26 (-0.24, 0.77)	0.31	0.25 (-0.27, 0.78)	0.34
rs822396	3	*ADIPOQ*	A/G	-0.12 (-0.68, 0.43)	0.66	0.30 (-0.28, 0.88)	0.31
rs2241766	3	*ADIPOQ*	T/G	-0.05 (-0.59, 0.48)	0.84	0.06 (-0.50, 0.62)	0.84
rs1501299	3	*ADIPOQ*	G/T	0.60 (0.10, 1.10)	0.02	-0.20 (-0.72, 0.33)	0.46
rs2232853	1	*ADIPOR1*	G/A	0.16 (-0.36, 0.67)	0.55	0.40 (-0.13, 0.94)	0.14
rs12733285	1	*ADIPOR1*	C/T	-0.49 (-1.03, 0.05)	0.08	-0.14 (-0.70, 0.43)	0.63
rs1342387	1	*ADIPOR1*	T/C	0.15 (-0.39, 0.69)	0.59	0.27 (-0.30, 0.84)	0.36
rs7539542	1	*ADIPOR1*	C/G	0.28 (-0.23, 0.78)	0.29	-0.04 (-0.58, 0.49)	0.87
rs10920531	1	*ADIPOR1*	C/A	0.57 (0.05, 1.09)	0.03	0.01 (-0.54, 0.56)	0.97
rs1029629	12	*ADIPOR2*	T/G	0.01 (-0.50, 0.52)	0.98	-0.20 (-0.73, 0.34)	0.47
rs7975600	12	*ADIPOR2*	A/T	-0.12 (-0.68, 0.44)	0.68	-0.22 (-0.81, 0.36)	0.45
rs11612383	12	*ADIPOR2*	G/A	-0.06 (-0.56, 0.44)	0.81	0.09 (-0.43, 0.61)	0.74
rs1058322	12	*ADIPOR2*	C/T	-0.05 (-0.55, 0.45)	0.83	-0.20 (-0.72, 0.33)	0.46
rs11061973	12	*ADIPOR2*	G/A	0.27 (-0.26, 0.81)	0.31	0.18 (-0.38, 0.74)	0.53
rs2108642	12	*ADIPOR2*	C/A	-0.22 (-0.79, 0.35)	0.45	-0.33 (-0.93, 0.27)	0.28
rs767870	12	*ADIPOR2*	A/G	-0.19 (-0.73, 0.36)	0.50	-0.14 (-0.71, 0.43)	0.63
rs12342	12	*ADIPOR2*	C/T	0.01 (-0.50, 0.52)	0.97	-0.03 (-0.56, 0.50)	0.90
rs1044471	12	*ADIPOR2*	C/T	0.17 (-0.40, 0.74)	0.56	0.16 (-0.44, 0.75)	0.60
rs7294540	12	*ADIPOR2*	C/A	0.14 (-0.38, 0.67)	0.59	-0.21 (-0.76, 0.34)	0.46

Chr., chromosome. SNP: single nucleotide polymorphisms. M/m indicates major/minor allele.

All models are adjusted for smoking.

**Table 3:** Associations of genetic variants with probability of being overweight or obese.

					Overweight		Obese	
SNP	Chr.	Position	Gene	M/m	OR (95% CI)	*P*-value	OR (95% CI)	*P*-value
rs266729	3	186559474	*ADIPOQ*	C/G	1.31 (0.95, 1.79)	0.10	1.15 (0.71, 1.88)	0.57
rs822395	3	186566807	*ADIPOQ*	A/C	0.97 (0.71, 1.33)	0.87	0.98 (0.60, 1.59)	0.93
rs822396	3	186566877	*ADIPOQ*	A/G	1.17 (0.83, 1.65)	0.36	0.97 (0.57, 1.67)	0.93
rs2241766	3	186570892	*ADIPOQ*	T/G	1.22 (0.88, 1.69)	0.23	1.08 (0.66, 1.83)	0.72
rs1501299	3	186571123	*ADIPOQ*	G/T	0.78 (0.57, 1.07)	0.13	0.84 (0.52, 1.38)	0.50
rs2232853	1	202931958	*ADIPOR1*	G/A	1.24 (0.90, 1.70)	0.19	1.61 (0.99, 2.61)	0.05
rs12733285	1	202922040	*ADIPOR1*	C/T	0.80 (0.58, 1.11)	0.19	0.91 (0.54, 1.53)	0.73
rs1342387	1	202914356	*ADIPOR1*	T/C	1.32 (0.93, 1.88)	0.12	1.03 (0.61, 1.74)	0.90
rs7539542	1	202909974	*ADIPOR1*	C/G	0.87 (0.64, 1.20)	0.40	1.14 (0.69, 1.87)	0.61
rs10920531	1	202908836	*ADIPOR1*	C/A	0.89 (0.64, 1.23)	0.48	1.11 (0.67, 1.86)	0.68
rs1029629	12	1799267	*ADIPOR2*	T/G	0.84 (0.61, 1.15)	0.28	0.86 (0.53, 1.41)	0.56
rs7975600	12	1815252	*ADIPOR2*	A/T	0.74 (0.51, 1.06)	0.10	0.90 (0.52, 1.55)	0.70
rs11612383	12	1831355	*ADIPOR2*	G/A	1.01 (0.74, 1.38)	0.95	1.10 (0.68, 1.78)	0.70
rs1058322	12	1836979	*ADIPOR2*	C/T	0.88 (0.64, 1.20)	0.41	0.78 (0.48, 1.26)	0.31
rs11061973	12	1865936	*ADIPOR2*	G/A	1.20 (0.86, 1.67)	0.28	1.21 (0.73, 2.01)	0.46
rs2108642	12	1866799	*ADIPOR2*	C/A	0.79 (0.56, 1.12)	0.18	0.82 (0.48, 1.40)	0.46
rs767870	12	1889823	*ADIPOR2*	A/G	0.79 (0.56, 1.12)	0.18	0.77 (0.45, 1.33)	0.35
rs12342	12	1896880	*ADIPOR2*	C/T	0.92 (0.67, 1.26)	0.60	1.10 (0.67, 1.80)	0.70
rs1044471	12	1896956	*ADIPOR2*	C/T	1.33 (0.92, 1.93)	0.13	1.00 (0.58, 1.73)	0.99
rs7294540	12	1899714	*ADIPOR2*	C/A	0.84 (0.60, 1.16)	0.28	0.81 (0.49, 1.33)	0.40

Models are adjusted for smoking status.

Chr., chromosome. Position SNP: single nucleotide polymorphisms. M/m indicates major/minor allele.

### Associations with lung function level

When considering lung function (Table 4) we observed an overall significant increase in FEV_1_% predicted with increasing BMI (β = 0.53, 95% CI: 0.27, 0.78), in FVC % predicted (β = 1.02, 95% CI: 0.73, 1.30), and a decrease in FEV_1_/FVC with increasing BMI (β = -0.53, 95% CI: -0.71, -0.35). The serum levels of adiponectin were not associated with FEV_1_% predicted (β = 0.07, 95% CI: -0.20, 0.34), nor with FVC % predicted (β = -0.15, 95% CI: -0.46, 0.16), though adiponectin levels were significantly associated with FEV_1_/FVC (β = 0.21, 95% CI: 0.02, 0.40).

**Table 4:** Association of BMI and adiponectin with lung function.

	FEV_1_% predicted		FVC % predicted		FEV_1_/FVC	
	β-coefficient	95% CI	β-coefficient	95% CI	β-coefficient	95% CI
**BMI (kg/m**^**2**^)*	0.53	0.27, 0.78	1.02	0.73, 1.30	-0.53	-0.71, -0.35
Adiponectin(ng/ml)**	0.07	-0.20, 0.34	-0.15	-0.46, 0.16	0.21	0.02, 0.40

BMI: Body Mass Index, FEV_1_: Forced expiratory volume at 1s, FVC: Forced expiratory volume

*adjusted for smoking

**adjusted for BMI and smoking

**Fig 1:**
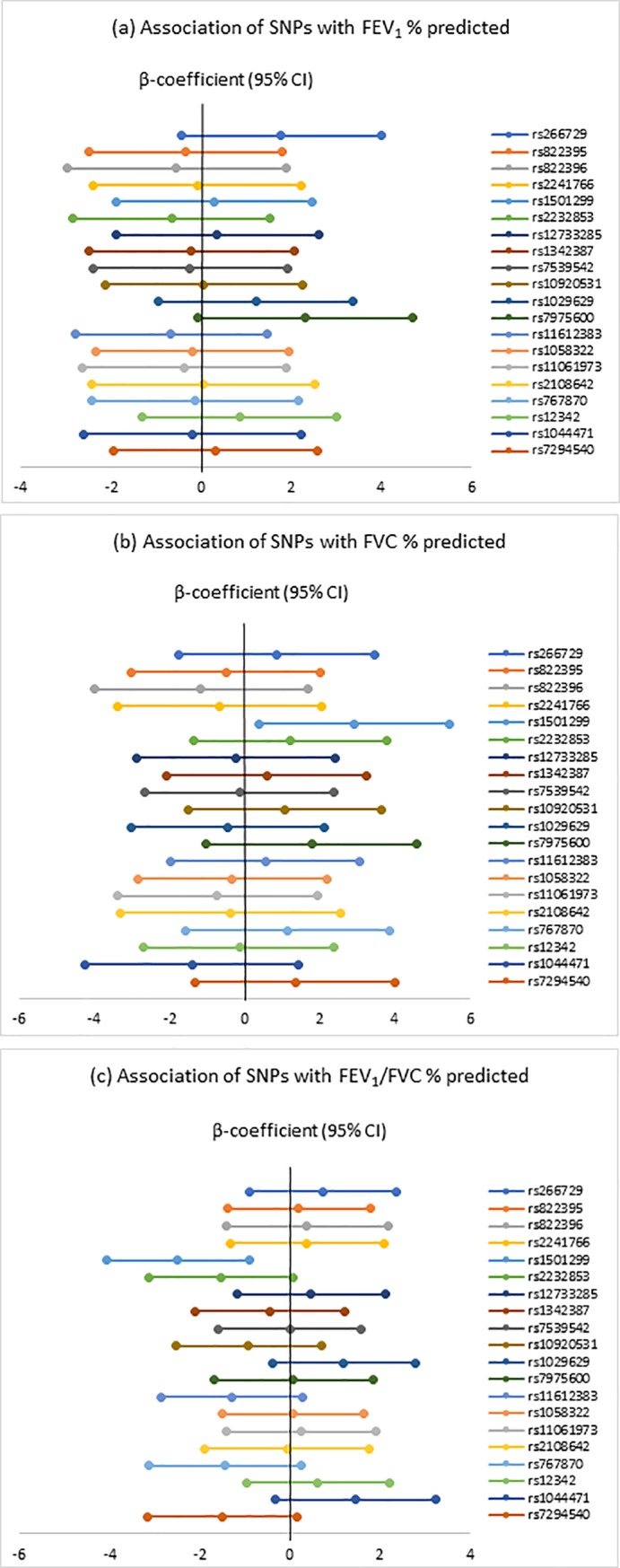
Association of genetic variants with FEV_1_% predicted, FVC % predicted and FEV_1_/FVC % predicted.

Fig 1 and S4 Table summarize the associations of SNPs in *ADIPOQ*, *ADIPOR1*, *ADIPOR2* with FEV_1_ and FVC % predicted, and FEV_1_/FVC. There was a borderline statistically significant association of rs7975600 in *ADIPOR2* with FEV_1_% predicted (β = 2.31, 95% CI: -0.07, 4.70, p = 0.06) and of rs2232853 in *ADIPOR1* with FEV_1_/FVC (β = -1.52, 95% CI: -3.12, 0.08, p = 0.06). In addition, rs1501299 in *ADIPOQ* was associated with FVC % predicted (β = 2.91, 95% CI: 0.38, 5.44, p = 0.02) and FEV_1_/FVC (β = -2.48, 95% CI: -4.05, -0.90, p<0.01), but not with FEV_1_% predicted (β = 0.30, 95% CI: -1.87, 2.46, p = 0.79).

## Discussion

The study examined the variation in three adiponectin-related genes in relation to adiponectin levels, BMI, lung function, and the risk of overweight and obesity in a sample of 18-year-old Cypriot men. We found a significant association between adiponectin levels and three SNPs in this population. The upstream *ADIPOQ* rs266729 was significantly associated with lower adiponectin while rs1501299 was associated with higher levels of adiponectin. Both the direction and associations of these two SNPs recapitulate that of previous candidate gene studies that evaluated these associations with adiponectin concentrations [21,22].

The SNPs considered to be functional in the present study were not significantly associated with BMI, however, rs266729 in *ADIPOQ* had a borderline association with the probability of being overweight, but not with the probability of being obese. This is in agreement with the findings from other studies, including the Framingham Offspring Study [23], the Heritage Family Study [24], the Oulu Diabetic Study [25], the Quebec Family Study of French-Canadians[26] and the more recent study by Cohen and colleagues [27]. Cohen et al. investigated 19 SNPs in *ADIPOR1* (including rs12733285, rs1342387, rs7539542, and rs10920531 genotyped in our study) and 27 SNPs in *ADIPOR2* (including rs12342, rs1044471, and rs7294540 also genotyped in our study) in relation to adiponectin levels and BMI and found no statistically significant associations [27]; we also observed no association of these SNPs with BMI but we obtained an association with adiponectin levels for rs10920531 in *ADIPOR1*. We noted that in the Cohen et al. study the relation between adiponectin levels and rs10920531 approached significance among black women but not among white women. Similarly the GEMS Study and the Diabetes Prevention Program Study found no genome-wide significant associations between adiponectin levels and SNPs in *ADIPOR1* in a GWAS of Europeans [28] and that of mixed race/ethnic groups of White, African American, Hispanic, Asian/Pacific Islanders, and American Indian ancestry [29], respectively. We speculate that one plausible reason for the discrepancy seen in the case of this SNP could be due to differences between race and gender. Interestingly, recent genome-wide studies have identified variants in a third adiponectin receptor, T-cadherin encoding CHD [13] that also affect circulating adiponectin levels, but unfortunately this information was not available in our study [28,30]. Similarly, while some studies have reported positive associations between BMI and some *ADIPOQ* polymorphisms [31–34] these were on other SNPs that were not available in our study.

We also had the opportunity to examine the association of BMI, adiponectin levels, and genetic variation with lung function measurements. The findings suggest that FEV_1_ and FVC % predicted in this age group increase with increasing BMI, while FEV_1_/FVC tends to decrease as BMI increases. Even though findings in adult populations suggest that lung function decreases with increasing BMI [8], our results are supported by studies in children aged 8 to 17 years old, showing that larger BMI and larger waist circumference are associated with higher FEV_1_ and FVC in both genders in children aged 8 to 17 [35] as their lungs are still growing. It is likely that the larger effect of BMI on FVC % predicted compared to FEV_1_% predicted results in lower FEV_1_/FVC % predicted in these children, a finding which is in line with previous studies [36,37].

Notably, rs266729 which was found to be associated with decreased adiponectin levels in our study, increases FEV_1_% predicted, while rs1501299 which is linked to increased adiponectin it is also associated with airflow obstruction (decreased FEV_1_/FVC). These findings are consistent with Thyagarajan et al. positively linking adiponectin levels with lung function[38]. However, the null association with BMI suggests that *ADIPOQ* and adiponectin levels may play a role in lung biologic and lung function level in young male individuals, independent of obesity.

Our study has several limitations as well as strengths. First, we analyzed data only from a group of 18-year old men and as gender is an important determinant of adiponectin levels the results from this study cannot be easily generalized to women or other ethnic or age groups. Furthermore, it was difficult to find a similar population (young 18 years old, males only, with genetic data, lung function and serum adipokines available) to run a replication study. In addition, the study analyzed total serum adiponectin levels measured at a single point in time which may not fully characterize long-term adiponectin exposures and effects; however, studies have shown adiponectin levels to be very reproducible, with adiponectin concentrations when measured repeatedly, on average one year apart, being highly correlated [39]. Another limitation is the possibility of false positive findings arising from the multiple comparisons made; however, the SNPs analyzed were those earlier known to be functionally relevant from a pool of SNPs tagging the two major haplotype blocks within each gene among Europeans. The few SNPs that were significantly associated with adiponectin levels in this study recapitulated results of earlier studies, thereby validating this approach. Moreover, this study was of limited power to detect modest differences between genotypes. The wide CIs in this study were due largely to the small sample size especially in the pairwise comparisons of BMI groups where the small number of people particularly in the obesity group was a limiting factor. Finally, the SNPs analyzed in the present study were not powered to detect rare variants; it could be that other genes exist, working in concert with adiponectin-related genes, which would need to be included to observe an effect and such would require a much larger sample size. A system genetics analysis of the molecular mechanisms underlying variations in lung function has revealed that SNPs associated with lung function were enriched for lung expression quantitative trait loci (eQTLs) [40]. In such study, a large number of SNPs that determine the variation in lung function measures were also enriched in developmental and inflammatory pathways. Interestingly, our study has identified SNPs within the *ADIPOQ* gene underlying variation in lung function independent of BMI in young Cypriot males, representing testable hypothesis for future GWAS and more sophisticated post-GWAS analyses including pathway and gene set enrichment analysis and ultimately, causality investigation using Mendelian randomization to estimate the influence of blood adiponectin on lung function and lung function risk factors in the Cypriot population.

One could suggest that the lack of multiple testing correction is responsible for the current results. We decided not to apply a sequential (classical) Bonferroni correction for a number of reasons. First, our choice for the current study was explicitly driven by previous observations suggesting adiponectin levels are associated with lung function. Secondly, the independent variables in our analyses (e.g. BMI and lung function) are related, indicating that a rigid statistical procedure, such as Bonferroni correction for multiple testing, would not do justice to their biologically linked nature. Finally, although adjustment for multiple testing will decrease the chance of a type I error, it will also increase the chance of a type II error, so that a true association is not found. This is especially possible in a relatively small study such as ours. Thus, we followed the advice given by Perneger [41]: “Simply describing what was done and why, and discussing the possible interpretations of each result, should enable the reader to reach a reasonable conclusion without the help of Bonferroni adjustments”.

In conclusion, the present findings confirmed and further strengthened the current knowledge regarding the role of adiponectin on lung function. This is the first study to link functional variants in *ADIPOQ* gene with lung function in young males and confirmed that adiponectin levels influence lung function independent of obesity. Further studies should concentrate on the role of adipokines on lung function which may direct novel therapeutic approaches.

## Supporting information

S1 Table Genotypic characteristics of 5 ADIPOQ SNPs, 5 ADIPOR1 SNPs, and 10 ADIPOR2 SNPs. (DOCX)

S2 Table Associations of genetic variants with adiponectin. (DOCX)

S3 Table Associations of genetic variants with Waist circumference, waist-to-height ratio, sum of skinfolds and fat percentage. (DOCX)

S4 Table Association of SNPs with lung function. (DOCX)

S1 Fig LD plots of SNPs within ADIPOQ, ADIPOR1, and ADIPOR2. (DOCX)

S1 Dataset Participants’ dataset. (SAS7BDAT)
