# Supplementary material for: Variants in ADIPOQ gene are linked to adiponectin levels and lung function in young males independent of obesity
Source: PLoS One. 2020 Jan 24;15(1):e0225662. doi: 10.1371/journal.pone.0225662 (PMC6980555; doi:10.1371/journal.pone.0225662)
Supplement: S1 Table — (DOCX) [file pone.0225662.s001.docx]

**S1 Table.** Genotypic characteristics of 5 *ADIPOQ* SNPs, 5 *ADIPOR1* SNPs, and 10 *ADIPOR2* SNPs.

|  |  |  | Allele | |  |  |  |  | Genotype count | | | | | |
| --- | --- | --- | --- | --- | --- | --- | --- | --- | --- | --- | --- | --- | --- | --- |
| SNP | Chr. | Gene | M | m | MAF (Obese) | MAF (Overweight) | MAF (Ref. weight) | MAF in HapMap-CEU^†^ | Obese (MM/Mm/mm) | P_HW_ | Overweight (MM/Mm/mm) | P_HW_ | Ref. weight (MM/Mm/mm) | P_HW_ |
| rs266729 | 3 | *ADIPOQ* | C | G | 0.230 | 0.242 | 0.209 | 0.308 | 44/26/4 | 0.768 | 120/80/11 | 0.710 | 428/229/29 | 0.908 |
| rs822395 | 3 | *ADIPOQ* | A | C | 0.278 | 0.290 | 0.280 | 0.354^a^ | 37/30/5 | 1.000 | 108/78/21 | 0.237 | 352/280/51 | 0.704 |
| rs822396 | 3 | *ADIPOQ* | A | G | 0.142 | 0.167 | 0.146 | 0.158^b^ | 54/19/1 | 0.685 | 145/60/5 | 0.808 | 499/170/15 | 0.879 |
| rs2241766 | 3 | *ADIPOQ* | T | G | 0.196 | 0.192 | 0.175 | 0.133^b^ | 49/21/4 | 0.325 | 135/71/5 | 0.272 | 472/190/25 | 0.290 |
| rs1501299 | 3 | *ADIPOQ* | G | T | 0.257 | 0.261 | 0.307 | 0.300^b^ | 38/31/3 | 0.374 | 114/81/14 | 1.000 | 327/300/61 | 0.532 |
| rs2232853 | 1 | *ADIPOR1* | G | A | 0.270 | 0.239 | 0.212 | 0.336 | 38/32/4 | 1.000 | 121/79/11 | 0.850 | 429/225/33 | 0.647 |
| rs12733285 | 1 | *ADIPOR1* | C | T | 0.453 | 0.386 | 0.440 | 0.301 | 24/33/17 | 0.275 | 73/112/25 | 0.082 | 212/342/130 | 0.756 |
| rs1342387 | 1 | *ADIPOR1* | T | C | 0.473 | 0.481 | 0.423 | 0.450 | 23/32/19 | 0.201 | 53/111/45 | 0.407 | 220/339/127 | 0.877 |
| rs7539542 | 1 | *ADIPOR1* | C | G | 0.372 | 0.327 | 0.342 | 0.288 | 28/37/9 | 0.811 | 94/96/21 | 0.754 | 285/331/69 | 0.062 |
| rs10920531 | 1 | *ADIPOR1* | C | A | 0.419 | 0.379 | 0.394 | 0.358 | 24/38/12 | 0.824 | 80/102/29 | 0.771 | 243/347/97 | 0.150 |
| rs1029629 | 12 | *ADIPOR2* | T | G | 0.349 | 0.330 | 0.358 | 0.292 | 33/29/11 | 0.340 | 94/92/23 | 1.000 | 279/314/86 | 0.934 |
| rs7975600 | 12 | *ADIPOR2* | A | T | 0.142 | 0.133 | 0.163 | 0.127 | 54/19/1 | 1.000 | 161/44/6 | 0.222 | 484/176/23 | 0.161 |
| rs11612383 | 12 | *ADIPOR2* | G | A | 0.311 | 0.301 | 0.310 | 0.323 | 34/34/6 | 0.617 | 102/91/18 | 0.870 | 332/283/71 | 0.372 |
| rs1058322 | 12 | *ADIPOR2* | C | T | 0.291 | 0.288 | 0.317 | 0.283 | 39/27/8 | 0.431 | 107/87/17 | 1.000 | 323/291/72 | 0.597 |
| rs11061973 | 12 | *ADIPOR2* | G | A | 0.199 | 0.197 | 0.171 | 0.167 | 47/23/3 | 1.000 | 134/66/8 | 1.000 | 467/200/17 | 0.500 |
| rs2108642 | 12 | *ADIPOR2* | C | A | 0.466 | 0.469 | 0.490 | 0.482 | 21/37/16 | 0.670 | 62/100/49 | 0.491 | 167/335/181 | 0.646 |
| rs767870 | 12 | *ADIPOR2* | A | G | 0.135 | 0.148 | 0.178 | 0.155 | 54/20/0 | 1.000 | 153/50/6 | 0.414 | 467/196/24 | 0.515 |
| rs12342 | 12 | *ADIPOR2* | C | T | 0.351 | 0.334 | 0.339 | 0.332 | 30/36/8 | 1.000 | 94/93/24 | 0.878 | 293/318/73 | 0.349 |
| rs1044471 | 12 | *ADIPOR2* | C | T | 0.473 | 0.481 | 0.488 | 0.478 | 20/37/16 | 1.000 | 45/112/53 | 0.407 | 186/328/170 | 0.285 |
| rs7294540 | 12 | *ADIPOR2* | C | A | 0.390 | 0.400 | 0.424 | 0.447 | 28/33/12 | 0.648 | 79/94/37 | 0.318 | 229/327/126 | 0.638 |

SNP: single nucleotide polymprphisms. M/m indicates major/minor allele. MAF indicates minor allelic frequency. P_HW_ indicates *P* value for Hardy-Weinberg equilibrium. ^†^MAF in HapMap-CEU indicates minor allele frequencies based on Central European (CEU) samples from International HapMap project samples of European (EUR) population or from ^a^Perlegen AFD_AFR panel EUR samples selected from the human variation panel of 50 Caucasians (HD50 CAU-Coriell Cell Repository) or from ^b^Pilot_1_CEU_low_coverage_panel, using the NCBI dbSNP (<https://www.ncbi.nlm.nih.gov/SNP/>).
