# Supplementary material for: Variants in ADIPOQ gene are linked to adiponectin levels and lung function in young males independent of obesity
Source: PLoS One. 2020 Jan 24;15(1):e0225662. doi: 10.1371/journal.pone.0225662 (PMC6980555; doi:10.1371/journal.pone.0225662)
Supplement: S2 Table — (DOCX) [file pone.0225662.s002.docx]

**S2 Table.** Associations of genetic variants with adiponectin

| SNP | Chr. | Position | Gene | M/m | β-coefficient | *P-value* |
| --- | --- | --- | --- | --- | --- | --- |
| rs266729 | 3 | 186559474 | *ADIPOQ* | C/G | -0.55 | 0.03 |
| rs822395 | 3 | 186566807 | *ADIPOQ* | A/C | 0.28 | 0.26 |
| rs822396 | 3 | 186566877 | *ADIPOQ* | A/G | -0.12 | 0.67 |
| rs2241766 | 3 | 186570892 | *ADIPOQ* | T/G | -0.07 | 0.79 |
| rs1501299 | 3 | 186571123 | *ADIPOQ* | G/T | 0.60 | 0.02 |
| rs2232853 | 1 | 202931958 | *ADIPOR1* | G/A | 0.22 | 0.39 |
| rs12733285 | 1 | 202922040 | *ADIPOR1* | C/T | -0.51 | 0.06 |
| rs1342387 | 1 | 202914356 | *ADIPOR1* | T/C | 0.23 | 0.41 |
| rs7539542 | 1 | 202909974 | *ADIPOR1* | C/G | 0.30 | 0.24 |
| rs10920531 | 1 | 202908836 | *ADIPOR1* | C/A | 0.63 | 0.02 |
| rs1029629 | 12 | 1799267 | *ADIPOR2* | T/G | -0.02 | 0.94 |
| rs7975600 | 12 | 1815252 | *ADIPOR2* | A/T | -0.14 | 0.61 |
| rs11612383 | 12 | 1831355 | *ADIPOR2* | G/A | -0.06 | 0.83 |
| rs1058322 | 12 | 1836979 | *ADIPOR2* | C/T | -0.09 | 0.72 |
| rs11061973 | 12 | 1865936 | *ADIPOR2* | G/A | 0.29 | 0.27 |
| rs2108642 | 12 | 1866799 | *ADIPOR2* | C/A | -0.27 | 0.34 |
| rs767870 | 12 | 1889823 | *ADIPOR2* | A/G | -0.20 | 0.46 |
| rs12342 | 12 | 1896880 | *ADIPOR2* | C/T | 0.01 | 0.98 |
| rs1044471 | 12 | 1896956 | *ADIPOR2* | C/T | 0.18 | 0.54 |
| rs7294540 | 12 | 1899714 | *ADIPOR2* | C/A | 0.11 | 0.67 |

Models were adjusted for ever smoking and BMI.
