# Supplementary material for: Variants in ADIPOQ gene are linked to adiponectin levels and lung function in young males independent of obesity
Source: PLoS One. 2020 Jan 24;15(1):e0225662. doi: 10.1371/journal.pone.0225662 (PMC6980555; doi:10.1371/journal.pone.0225662)
Supplement: S3 Table — (DOCX) [file pone.0225662.s003.docx]

**S3 Table.** Associations of genetic variants with Waist circumference, waist-to-height ratio, sum of skinfolds and fat percentage

|  |  |  |  |  | Waist circumference | | Waist-to-Height | | Skinfolds | | Fat percentage | |
| --- | --- | --- | --- | --- | --- | --- | --- | --- | --- | --- | --- | --- |
| SNP | Chr. | Position | Gene | M/m | β-coefficient | *P*-value | β-coefficient | *P*-value | β-coefficient | *P*-value | β-coefficient | *P*-value |
| rs266729 | 3 | 186559474 | *ADIPOQ* | C/G | 0.57 | 0.42 | 5.5*10^-3^ | 0.16 | 2.17 | 0.18 | 0.46 | 0.30 |
| rs822395 | 3 | 186566807 | *ADIPOQ* | A/C | 0.27 | 0.69 | 3.6*10^-3^ | 0.34 | 1.17 | 0.46 | 0.47 | 0.27 |
| rs822396 | 3 | 186566877 | *ADIPOQ* | A/G | -0.03 | 0.97 | 1.3*10^-3^ | 0.76 | 1.10 | 0.53 | 0.43 | 0.37 |
| rs2241766 | 3 | 186570892 | *ADIPOQ* | T/G | 0.37 | 0.62 | -1.0*10^-3^ | 0.81 | 0.96 | 0.57 | 0.04 | 0.94 |
| rs1501299 | 3 | 186571123 | *ADIPOQ* | G/T | -0.20 | 0.77 | -1.0*10^-3^ | 0.80 | -0.92 | 0.56 | -0.02 | 0.96 |
| rs2232853 | 1 | 202931958 | *ADIPOR1* | G/A | 1.33 | 0.06 | 6.7*10^-3^ | 0.08 | 3.04 | 0.06 | 0.67 | 0.13 |
| rs12733285 | 1 | 202922040 | *ADIPOR1* | C/T | -0.22 | 0.77 | -1.8*10^-3^ | 0.67 | -1.59 | 0.35 | -0.27 | 0.55 |
| rs1342387 | 1 | 202914356 | *ADIPOR1* | T/C | 0.83 | 0.26 | 4.5*10^-3^ | 0.27 | 1.65 | 0.33 | 0.43 | 0.35 |
| rs7539542 | 1 | 202909974 | *ADIPOR1* | C/G | -0.41 | 0.55 | -2.9*10^-3^ | 0.45 | -0.25 | 0.88 | -0.06 | 0.89 |
| rs10920531 | 1 | 202908836 | *ADIPOR1* | C/A | -0.11 | 0.87 | -2.1*10^-3^ | 0.60 | 0.01 | 0.99 | -0.17 | 0.70 |
| rs1029629 | 12 | 1799267 | *ADIPOR2* | T/G | -0.72 | 0.30 | -4.1*10^-3^ | 0.28 | -0.67 | 0.67 | -0.17 | 0.70 |
| rs7975600 | 12 | 1815252 | *ADIPOR2* | A/T | -1.05 | 0.17 | -2.7*10^-3^ | 0.53 | -1.53 | 0.39 | -0.16 | 0.74 |
| rs11612383 | 12 | 1831355 | *ADIPOR2* | G/A | 0.40 | 0.56 | 0.4*10^-3^ | 0.91 | 0.87 | 0.58 | 0.01 | 0.97 |
| rs1058322 | 12 | 1836979 | *ADIPOR2* | C/T | -0.52 | 0.45 | -2.0*10^-3^ | 0.59 | -1.31 | 0.40 | -0.22 | 0.60 |
| rs11061973 | 12 | 1865936 | *ADIPOR2* | G/A | 0.61 | 0.41 | 1.5*10^-3^ | 0.70 | 1.25 | 0.46 | 0.17 | 0.71 |
| rs2108642 | 12 | 1866799 | *ADIPOR2* | C/A | -0.84 | 0.29 | -5.0*10^-3^ | 0.25 | -1.11 | 0.54 | -0.30 | 0.54 |
| rs767870 | 12 | 1889823 | *ADIPOR2* | A/G | 0.30 | 0.69 | 0.1*10^-3^ | 0.98 | -0.06 | 0.97 | -0.05 | 0.92 |
| rs12342 | 12 | 1896880 | *ADIPOR2* | C/T | -0.34 | 0.63 | -1.2*10^-3^ | 0.76 | 0.44 | 0.78 | 0.07 | 0.87 |
| rs1044471 | 12 | 1896956 | *ADIPOR2* | C/T | 0.19 | 0.80 | 1.4*10^-3^ | 0.74 | 1.34 | 0.46 | 0.18 | 0.71 |
| rs7294540 | 12 | 1899714 | *ADIPOR2* | C/A | -0.29 | 0.69 | -1.5*10^-3^ | 0.70 | -0.26 | 0.87 | -0.18 | 0.69 |

Models were adjusted for ever smoking.
