# Supplementary material for: Variants in ADIPOQ gene are linked to adiponectin levels and lung function in young males independent of obesity
Source: PLoS One. 2020 Jan 24;15(1):e0225662. doi: 10.1371/journal.pone.0225662 (PMC6980555; doi:10.1371/journal.pone.0225662)
Supplement: S4 Table — (DOCX) [file pone.0225662.s004.docx]

**S4 Table.** Association of SNPs with lung function

|  |  |  | Allele | | FEV_1_ % predicted | | | FVC % predicted | | | FEV_1_/FVC % predicted | | |
| --- | --- | --- | --- | --- | --- | --- | --- | --- | --- | --- | --- | --- | --- |
| SNP | Chr. | Gene | M | m | β-coefficient | *P*-value | 95 % CI | β-coefficient | *P*-value | 95 % CI | β-coefficient | *P*-value | 95 % CI |
| rs266729 | 3 | *ADIPOQ* | C | G | 1.78 | 0.11 | (-0.43, 3.99) | 0.85 | 0.52 | (-1.74, 3.45) | 0.74 | 0.37 | (-0.89, 2.37) |
| rs822395 | 3 | *ADIPOQ* | A | C | -0.34 | 0.75 | (-2.49, 1.80) | -0.50 | 0.70 | (-3.01, 2.01) | 0.20 | 0.80 | (-1.38, 1.79) |
| rs822396 | 3 | *ADIPOQ* | A | G | -0.54 | 0.66 | (-2.97, 1.89) | -1.16 | 0.43 | (-4.01, 1.69) | 0.38 | 0.67 | (-1.40, 2.17) |
| rs2241766 | 3 | *ADIPOQ* | T | G | -0.08 | 0.94 | (-2.39, 2.23) | -0.66 | 0.63 | (-3.37, 2.05) | 0.39 | 0.66 | (-1.31, 2.08) |
| rs1501299 | 3 | *ADIPOQ* | G | T | 0.30 | 0.79 | (-1.87, 2.46) | 2.91 | 0.02 | (0.38, 5.44) | -2.48 | <0.01 | (-4.05, -0.90) |
| rs2232853 | 1 | *ADIPOR1* | G | A | -0.65 | 0.56 | (-2.84, 1.53) | 1.22 | 0.35 | (-1.35, 3.78) | -1.52 | 0.06 | (-3.12, 0.08) |
| rs12733285 | 1 | *ADIPOR1* | C | T | 0.36 | 0.75 | (-1.88, 2.61) | -0.23 | 0.86 | (-2.87, 2.41) | 0.48 | 0.57 | (-1.17, 2.13) |
| rs1342387 | 1 | *ADIPOR1* | T | C | -0.21 | 0.86 | (-2.48, 2.06) | 0.59 | 0.66 | (-2.07, 3.25) | -0.43 | 0.61 | (-2.10, 1.23) |
| rs7539542 | 1 | *ADIPOR1* | C | G | -0.24 | 0.82 | (-2.39, 1.91) | -0.14 | 0.91 | (-2.67, 2.38) | 9x10^-4^ | 0.99 | (-1.58, 1.58) |
| rs10920531 | 1 | *ADIPOR1* | C | A | 0.06 | 0.96 | (-2.13, 2.26) | 1.07 | 0.41 | (-1.50, 3.65) | -0.91 | 0.27 | (-2.52, 0.70) |
| rs1029629 | 12 | *ADIPOR2* | T | G | 1.22 | 0.27 | (-0.95, 3.38) | -0.45 | 0.73 | (-3.00, 2.11) | 1.20 | 0.14 | (-0.39, 2.80) |
| rs7975600 | 12 | *ADIPOR2* | A | T | 2.31 | 0.06 | (-0.07, 4.70) | 1.79 | 0.21 | (-1.01, 4.58) | 0.08 | 0.93 | (-1.68, 1.84) |
| rs11612383 | 12 | *ADIPOR2* | G | A | -0.66 | 0.54 | (-2.79, 1.48) | 0.56 | 0.66 | (-1.95, 3.07) | -1.28 | 0.11 | (-2.84, 0.28) |
| rs1058322 | 12 | *ADIPOR2* | C | T | -0.18 | 0.87 | (-2.32, 1.96) | -0.33 | 0.80 | (-2.84, 2.19) | 0.07 | 0.93 | (-1.50, 1.64) |
| rs11061973 | 12 | *ADIPOR2* | G | A | -0.36 | 0.75 | (-2.62, 1.90) | -0.72 | 0.59 | (-3.37, 1.93) | 0.26 | 0.76 | (-1.40, 1.92) |
| rs2108642 | 12 | *ADIPOR2* | C | A | 0.06 | 0.96 | (-2.42, 2.54) | -0.38 | 0.80 | (-3.29, 2.54) | -0.06 | 0.95 | (-1.89, 1.76) |
| rs767870 | 12 | *ADIPOR2* | G | A | -0.13 | 0.91 | (-2.43, 2.17) | 1.14 | 0.41 | (-1.57, 3.84) | -1.44 | 0.09 | (-3.13, 0.25) |
| rs12342 | 12 | *ADIPOR2* | C | T | 0.86 | 0.43 | (-1.30, 3.02) | -0.14 | 0.91 | (-2.68, 2.39) | 0.62 | 0.44 | (-0.96, 2.21) |
| rs1044471 | 12 | *ADIPOR2* | C | T | -0.19 | 0.88 | (-2.60, 2.22) | -1.40 | 0.33 | (-4.23, 1.43) | 1.46 | 0.10 | (-0.31, 3.23) |
| rs7294540 | 12 | *ADIPOR2* | A | C | 0.32 | 0.78 | (-1.94, 2.59) | 1.35 | 0.32 | (-1.30, 4.01) | -1.49 | 0.08 | (-3.15, 0.17) |

Models were adjusted for ever smoking.
