# Supplementary material for: Variants in ADIPOQ gene are linked to adiponectin levels and lung function in young males independent of obesity
Source: PLoS One. 2020 Jan 24;15(1):e0225662. doi: 10.1371/journal.pone.0225662 (PMC6980555; doi:10.1371/journal.pone.0225662)
Supplement: S1 Fig — (DOCX) [file pone.0225662.s005.docx]

**S1 Figure.** LD plots of SNPs within *ADIPOQ*, *ADIPOR1*, and *ADIPOR2.*

1. **ADIPOQ (B) ADIPOR1**


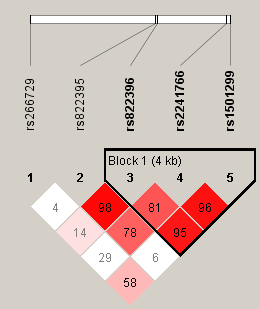

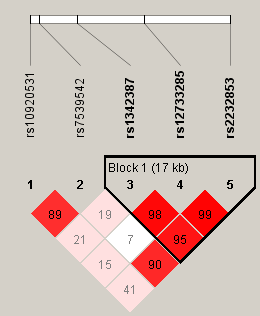


1. **ADIPOR2**


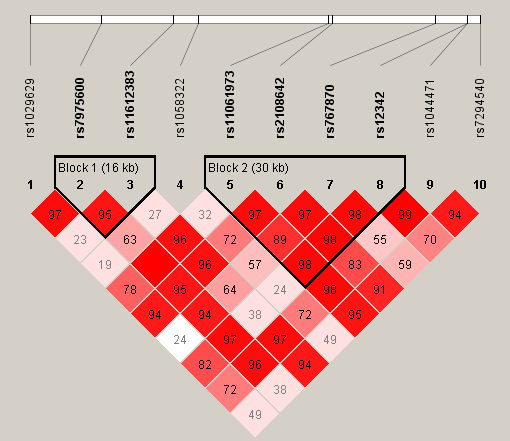


Pairwise LD among SNPs and haplotype blocks across *ADIPOQ* (A), *ADIPOR1* (B), and *ADIPOR2* (C). The D´ values for each SNP pair are shown. The lines connect each SNP name with the corresponding cell in the LD matrix. Increasing levels of LD are shown by darker red scale.
